# Effects of the COVID-19 Pandemic on Social Determinants of Mental Health and Psychiatric Decompensation

**DOI:** 10.1192/j.eurpsy.2022.1343

**Published:** 2022-09-01

**Authors:** J. Jay, E. Garrels, P. Korenis

**Affiliations:** 1 BronxCare Health System, Psychiatry, Bronx, United States of America; 2 Bronxcare Health System, Psychiatry, New York, United States of America

**Keywords:** pandemic, Covid-19, decompensation, Psychosis

## Abstract

**Introduction:**

The impact of the COVID-19 pandemic has propelled changes in healthcare delivery, incorporating new technologies and resulting in interruptions of care and access to treatment.

**Objectives:**

To understand the ways that the COVID-19 pandemic has affected mental health, particularly in those with psychotic disorders. The unique nature and scale of the COVID-19 pandemic today presents an opportunity to learn more about the challenges faced by our patients and improvements that can be made in the delivery of mental healthcare.

**Methods:**

We report five cases of patients with preexisting psychotic disorders seen on an inpatient psychiatry unit who decompensated for reasons relating to the COVID-19 pandemic. We conducted a review of the literature by searching the PubMed database for the keywords “mental health,” “psychosis,” “COVID-19,” “epidemic,” “pandemic,” and “coronavirus.

**Results:**

The prevalence of psychotic disorders in the US is estimated to be between 0.25% and 0.64%. In the context of an epidemic or pandemic, the incidence of psychotic symptoms in those infected with a virus is estimated to be between 0.9% and 4%, demonstrating increased risk to this group. The effects of the COVID-19 pandemic have contributed to psychiatric decompensation.

**Conclusions:**

The COVID-19 pandemic is an opportunity to identify ways in which our patients are at risk and how we can attempt to alleviate those risks to provide improved care going forward. By appreciating the multifaceted ways in which the current situation has affected our patient population, we can extrapolate lessons that will allow us to better serve our patients even when this pandemic passes.

**Disclosure:**

No significant relationships.

